# Ethanolic extract of okra has a potential gastroprotective effect on acute gastric lesions in Sprague Dawley rats

**DOI:** 10.1002/fsn3.1963

**Published:** 2020-10-23

**Authors:** Hafsa Yasin, Farwa Tariq, Aysha Sameen, Nazir Ahmad, Muhammad Faisal Manzoor, Maria Yasin, Tayyaba Tariq, Muhammad Waheed Iqbal, Bushra Ishfaq, Sana Mahmood, Azhari Siddeeg

**Affiliations:** ^1^ National Institute of Food Science and Technology Faculty of Food Nutrition and Home Sciences University of Agriculture Faisalabad Pakistan; ^2^ Institute of Home and Food Sciences Faculty of Life Sciences Government College University Faisalabad Pakistan; ^3^ School of Food Science and Engineering South China University and Technology Guangzhou China; ^4^ School of Food and Biological Engineering Jiangsu University Zhenjiang China; ^5^ District Head Quarters Hospital Pakpattan Pakistan; ^6^ Department of Food Engineering and Technology Faculty of Engineering and Technology University Gezira Wad Medani Sudan

**Keywords:** acute gastric damage, gastric pH, okra extract, phytochemicals, ulcer index

## Abstract

Okra (*Abelmoschus esculentus)* has various bioactive components used for the treatment of different diseases such as gastritis and ulcers. This research aims to examine the anti‐inflammatory and anti‐ulcer attributes of okra extract against gastric lesions. Adult Sprague Dawley male albino rats were divided into five groups. The negative control (G1) received normal feed, positive control (G2) received ulcer‐inducing drug aspirin 150 mg/kg of body weight (b.w), G3 group received reference drug omeprazole 20 mg/kg of b.w, G4 group received okra extract 250 mg/kg of b.w, and G5 group received 500 mg/kg of b.w. Acute gastric damage was induced in G1, G2, G3, and G4 using aspirin 150 mg/kg of b.w, during 14‐day‐long efficacy trials after that all the animals were sacrificed. Anti‐ulcer parameters and histopathological analysis of stomachs were performed to evaluate the degree of recovery against tissue damage by the administration of okra extract. The obtained results indicated that the 500 mg/kg of b.w okra extract exerted a protective effect in aspirin‐induced gastric ulcers by significantly (*p* < .05) reducing ulcer score, ulcer area, total acidity, and gastric volume, and significantly (*p *< .05) increasing gastric pH. Moreover, histopathological observation revealed that gastric mucosa was normal in G1, G3, G4, and G5; however, disruptions in the gastric epithelium were observed in G2. Congestion was observed in all groups except G1 and G5. Gastric pits and gastric glands were increased in size in G2 and G4. A higher concentration of okra extract (500 mg/kg of b.w) showed almost similar results when compared to the reference drug omeprazole.

## Graphical abstract

The biochemical and histopathological explorations indicate that okra Abelmoschus esculentus has potential gastroprotective against aspirin‐induced acute gastric lesions due to the presence of bioactive components. Okra helped in reducing congestion, irregularity in the muscular layer, increased gastric pits size, gastric ulcer area, ulcer scores, and ulcer area. Okra extract dose of 500 mg/Kg of body weight showed better results than 250 mg/Kg of body weight. Reduction in gastric ulcer by 500 mg/Kg of body weight was comparable to drug omeprazole. However, more advanced research is required to highlight the fundamental mechanism of the gastroprotective potential of okra, to get more advantageous and fruitful outcomes in this respect.

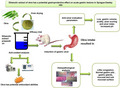

## 1 INTRODUCTION

Gastric ulcers are biological changes in the normal architecture of cells of the stomach wall that are caused by any erosion or disruption and get worse by persistent inflammation and oxidative stress. Although the basic mechanism is still under experimentation, it is considered that gastric ulcerations occur due to an imbalance between protective and damaging factors (Ardalani et al., 2020). The main prevalent causes of gastric ulcers are *Helicobacter pylori* bacteria, nonsteroidal anti‐inflammatory drugs, oxidative stress, genetic factors, alcohol consumption, and smoking (Park et al., 2020). If an ulcer is not properly treated, then gastric ulceration can lead to complications such as perforation of the stomach, bleeding, and even mortality (Ray et al., 2020).

Traditional methods used for treating gastric ulcers involve the restoration of the natural cytoarchitecture of the mucosal layer by the inhibition of damaging factors. These strategies include the usage of proton pump inhibitors, H_2_‐receptor antagonists, prostaglandin analogs, M1‐receptor blockers, and histamine‐2 receptor antagonists; modification of dietary habits; and lifestyle. Long‐term usage of drugs causes many side effects including gynecomastia, arrhythmia, hematopoietic changes, and impotence, and also recurrence of ulcer (Scally et al., 2018).

Medicinal plants have been used since prehistoric times to treat different types of diseases, due to the presence of bioactive components (Inoue et al., 2019). Any part of these medicinal plants (fruit, seeds, roots, and flower) can be used for disease treatment (Gray et al., 2003). Phenolics and flavonoids present in plants reduce oxidative stress and inflammation, and help to regenerate the mucosal layer after injury (Shahidi & Yeo, 2018).

Okra, scientifically known as *Abelmoschus esculentus*, from the family Malvaceae is cultivated in subtropical as well as tropical and warm areas (Kumar et al., 2013). Okra seeds, pods, leaves, fruit, even bud, and stem can be used for therapeutic purposes (Yonas et al., 2014). Okra is abundant in functional components such as lipid, mineral, carbohydrate, dietary fiber, amino acid, and vitamin (folate, niacin, choline, A, K, E, β‐carotene).

It also contains health‐promoting phenolics and flavonoids such as quercetin 30‐O‐xylosyl glucoside, quercetin 30‐O‐(600‐O‐malonyl)‐glucoside, epigallocatechin, quercetin 30‐O‐glucoside, myricetin, hibifolin, isoquercetin, hyperin, and rutin (Islam, 2019).

Okra is considered to exhibit antispasmodic, emollient poultice, diuretic, demulcent, laxative, cordial, and anticancer effects (van Dam et al., 2013). Moreover, in vitro and in vivo antidiabetic, antifatigue, antioxidant, antimicrobial, antihyperlipidemic, neuroprotective, and hepatoprotective impacts of okra have been described (Gemede et al., 2016; Xia et al., 2015). Quercetin has free radical scavenging ability toward superoxide anions, peroxyl, and hydroxyl radicals (Abourehab et al., 2015). Quercetin accelerated wound closure in cell scratch assay, reduced pro‐inflammatory cytokine production, and immune cell infiltration. Quercetin can suppress MAPK activation but not the NF‐κB pathway for regulation of inflammatory response in the pressure ulcer model (Yin et al., 2018).

Even though the medicinal impacts of okra extract on human wellness have not yet been adequately studied on gastric mucosal injury in experimental researches, keeping in view the global burden of gastric ulcers and the bioactive potential of okra, the present study is designed to assess the therapeutic efficacy of okra extract on acute gastric lesions in the rat model. Extract of whole okra fruit was used in the present study as it is consumed as a whole fruit rather than purified components.

## 2 MATERIALS AND METHODS

### 2.1 Materials

Okra (Abelmoschus esculentus) variety “Sabaz Pari” was procured from Ayub Agricultural Research Institute, Faisalabad, Pakistan. Aspirin (*Disprin* ^®^) and omeprazole were bought from a chemical supplier.

### 2.2 Preparation of extract

Fresh okra pods were washed and dried in a dehydrator at 55–60°C for 24 hr. After drying, grinding was done and okra powder was passed through a 20‐µm mesh size sieve and stored in polythene bags placed in an airtight jar. Overnight extraction was carried out by using 96% ethanol, and the extract was concentrated by using a rotary evaporator at 40°C (Alqasoumi, 2012). The percentage yield obtained was 13% w/w.

### 2.3 Antioxidant analysis of okra extract

Ethanolic extract of okra was subjected to antioxidant analysis by assessing total phenolic content (TPC) with the Folin–Ciocalteu method (Manzoor et al., 2019) and free radical scavenging activity through DPPH assay (Manzoor et al., 2020). Quantification of quercetin was done through high‐performance liquid chromatography by using column Shim‐Pack CLC‐ODS (C‐18), 25 cm 4.6 mm, 5 μm, and UV‐Vis detector. Gradient solvent system was used as the mobile phase: A (water:acetic acid; 94:6 pH = 2.27) and B (100% acetonitrile + water) at a flow rate of 1 ml/min. Absorbance was recorded at 280 nm (Careri et al., 2003).

### 2.4 Efficacy trial

Adult Sprague Dawley rats with an average weight of 167.43 g were selected and divided into five groups each containing four rats. Permission was taken from the Institutional Bioethical Committee of the University of Agriculture, Faisalabad, Pakistan. The animal trial was conducted by following the Principles of Laboratory Animal Care (Ed, 2011) Animals were kept in an animal room having room temperature about 22 ± 2°C, with 12/12 day and night cycle with proper ventilation. The treatment plan is given in Table 1. Animal feed was provided twice a day with free excess to tap water. Ulcer induction was done by using aspirin 150 mg/kg body weight (b.w) of animals for 14 days along with the normal feed. Anti‐ulcer drug omeprazole with the dosage of 20 mg/kg b.w of animals was fed as a reference drug to group 2 animals for comparison. Drugs and okra extract were administered orally by using an intragastric tube. After 14 days of administration of treatment material, all subjects were kept fasted for about 24 hr provided with free excess to water. Then, all subjects were decapitated and stomachs were dissected and preserved for further analysis.

**TABLE 1:** The treatment plan for an efficacy trial

Groups	Treatments
G1 (Negative control)	Normal feed
G2 (Positive control)	Normal feed + ulcer‐inducing drug aspirin (150 mg/kg body weight)
G3	Normal feed + reference drug (omeprazole) 20 mg/kg body weight
G4	Normal feed + 250 mg/kg body weight of okra extract
G5	Normal feed + 500 mg/kg body weight of okra extract

### 2.5 Anti‐ulcer analysis

Rat stomachs were opened along the greater curvature, cleaned, and washed gently using normal saline water to remove blood clots and gastric content and examined by a 10X magnifier lens to assess ulcer formation. Gastric volume was determined by centrifugation of gastric juice for 10 min at 1000rpm, and then, the volume of supernatant was noted. Total acid and pH values were assessed using pH meter and titration method, respectively (Raju et al., 2009). The ulcer area was measured in square millimeters (mm^2^), using a simple microscope (Wang et al., 1989). The extent of the ulcer was assessed by using ulcer score parameters (Raji et al., 2011). Ulcer index was calculated by adding the total number of ulcers per stomach and the mean of the severity of ulcer per stomach (Laine & Weinstein, 1988). Histopathological evaluation of gastric tissues was performed to see the recovery due to okra extract (Laine & Weinstein, 1988).

### 2.6 Statistical analysis

The calculated readings of control and treatment groups were compared using a one‐way analysis of variance (ANOVA) and Tukey's post‐test. SPSS (version 25) statistical program (SPSS Inc.) was used to carry out a one‐way analysis of variance (ANOVA) on acquired readings. A value of *p* < .05 was considered significant. Multiple mean comparisons were done using Tukey's post‐test to check either difference between means was significant or not. The mean and the standard deviation were used to express values.

## 3 RESULTS AND DISCUSSION

### 3.1 Antioxidant analysis of okra extract

The concentration of TPC in okra extract was found to be 141.37 ± 1.16 GAE/100 g (Table 2). This finding is comparable with the results reported that TPC was 142.48 ± 0.02 mg GAE/100 g and 10.75 ± 0.02 mg GAE/100 g in okra seeds and pulp, respectively (Khomsug et al., 2010). DPPH value in okra extract was 52.72 ± 0.80%. A study conducted to assess the free radical scavenging potential of total phenolic extract (TPE) of okra and its isolated phenolic compounds reported that the scavenging activity of TPE is 44.1%. This value is lower than the DPPH value recorded in the present study. This may be due to differences in variety, cultivation conditions, and environment (Hu et al., 2014). Quercetin amount observed was 37 mg/100 g in okra extract. Arapitsas (2008) reported that the amount of quercetin was 0.3 mg/g in okra skin. In the present research, amount of quercetin is slightly higher because the analysis was performed for whole okra fruit extract.

**TABLE 2:** Analysis of okra extract

Parameter	Mean ± *SD*
Total phenolic content	141.37 ± 1.16 GAE/100 g
DPPH	52.72 ± 0.80%
Quercetin	37 mg/100 g

### 3.2 Induction of gastric ulcer

Aspirin, a nonsteroid anti‐inflammatory drug (NSAID) 150 mg/kg of body weight, was used to induce stomach ulcers in the present study. Cyclooxygenase 1 (COX‐1) present in stomach mucosa is responsible for the production of gastroprotective prostaglandins. Aspirin being a potent ulcerogenic drug inhibits the activity of COX‐1 resulting in the suppression of protective prostaglandins. Moreover, it decreases bicarbonate secretion and gastric mucosal blood flow making gastric mucosa more susceptible to gastric mucosal injury and ulceration (Voutilainen et al., 2001). Numerous studies have demonstrated the aspirin‐induced gastric ulcers (Adefisayo et al., 2018; Moharram et al., 2017). Omeprazole is irreversible and selective proton pump inhibitor that was used as a reference anti‐ulcer drug. It inhibits the H+/K+‐ATPase system and gastric mucosa carbonic anhydrase enzyme. Consequently, gastric acid production is reduced leading to reduced ulceration (Adefisayo et al., 2018).

### 3.3 Anti‐ulcer evaluation parameters

#### 3.3.1 Gastric volume

**FIGURE 1:**
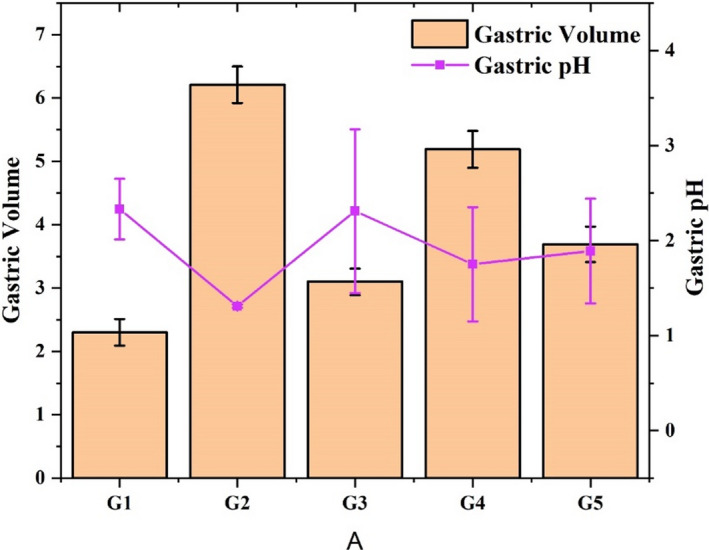
Mean gastric volume (ml) and gastric pH

Gastric volume significantly (*p *˂ 0.01) increased in aspirin‐administered rats (6.21 ± 0.29 ml) than the control group (2.30 ±** **0.21 ml) as shown in Figure 1. Reduction in gastric volume by the consumption of omeprazole (3.10 ± 0.21 ml) and 500 mg/kg b.w of okra extract (3.69 ± 0.28 ml) was nonsignificantly different from each other. A study that was conducted to investigate the anti‐ulcer effect of newly formulated drug “ranitidine mucoadhesive hydrogel formulae” demonstrated the increase in gastric volume due to ulcer lesions. Drug‐administered rats showed a reduction in gastric volume after reduced gastric ulcers than the control group (Arumugam et al., 2011).

#### 3.3.2 Gastric pH

The gastric pH of stomach contents significantly (*p* ˂ 0.01) decreased after the induction of gastric ulcers. Gastric pH of G1 (2.33 ± 0.320) and G3 (2.31 ± 1.860) was nonsignificantly (*p* > .05) different from each other. Omeprazole improved stomach pH in G3 as compared to the positive control group G2 (1.31 ± 0.017). It correlates with the study of Bhajoni et al. (2016) who assessed the anti‐ulcer effect of aqueous extract of leaves of *A. indica* on gastric ulcer using gastric pH as a biomarker. Extract of *A. indica* helped in attenuating raised gastric pH due to aspirin, pyloric ligation, and cold‐restraint stress‐induced gastric ulcer and exhibited potential anti‐ulcer properties.

#### 3.3.3 Total acidity

**FIGURE 2:**
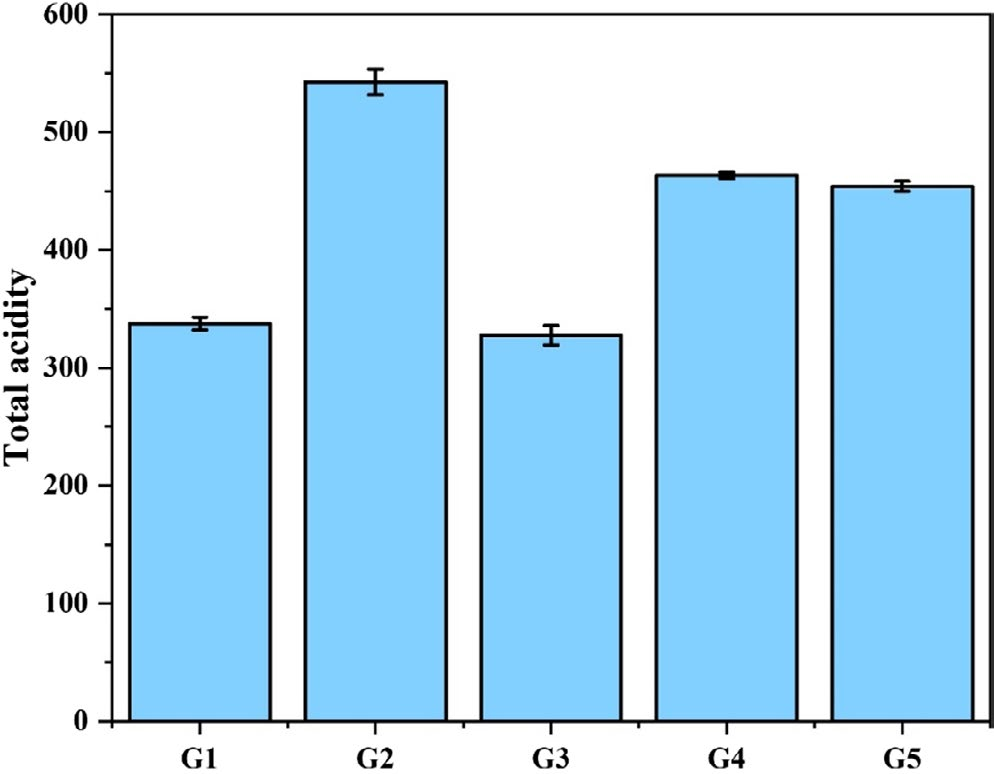
Mean total acidity of stomach

The lowest total acidity was observed in G3 than in other groups as presented in Figure 2. But this total acidity difference in G3 and G1 was nonsignificant (*p *> .05). The highest total acidity was observed in positive control group G2 (542.53 ± 10.78), while the G4 (463.63 ± 2.68) and G5 (454.07 ± 4.31) were found nonsignificantly (*p *> .05) different from each other. Similar findings were observed by Arumugam et al. (2011), who reported that total acidity increased after ulcer induction by the administration of ethanol and reduced after the anti‐ulceration effect of *Samanea saman (Jacq)* merr bark. Bhajoni et al. (2016) observed a significant reduction in total acidity after the treatment of ulcers with leaves of *A. indica*. Gastrin hormone is released by G cells present in the gastric antrum. Gastrin acts on the enterochromaffin‐like cells in the gastric corpus to release histamine, which stimulates parietal cells to secrete acid. Parietal cells are also directly stimulated by gastrin and it also promotes the growth of parietal and enterochromaffin‐like cells (Calam & Baron, 2001). Infection impairs the acid‐mediated inhibitory control responsible for gastrin and ultimately gastrin secretion is increased stimulating excessive acid secretion leading to ulceration (McColl et al., 2000). Increased production of gastric juice results in more gastric ulcers, acidity, ulcer scoring, ulcer area, and ulcer index and declined gastric pH. The gastric acid inhibiting ability of okra extract may be as a result of a direct action of lectins on cells involved in acid production. Lectins are the glycoproteins having the ability to recognize and bind carbohydrates (Bardocz et al., 1995) are present in okra (Caluête et al., 2015). They can inhibit the uptake of aminopyrine by parietal cells (Healey et al., 1998).

#### 3.3.4 Ulcer scores, ulcer area, and ulcer index

**FIGURE 3:**
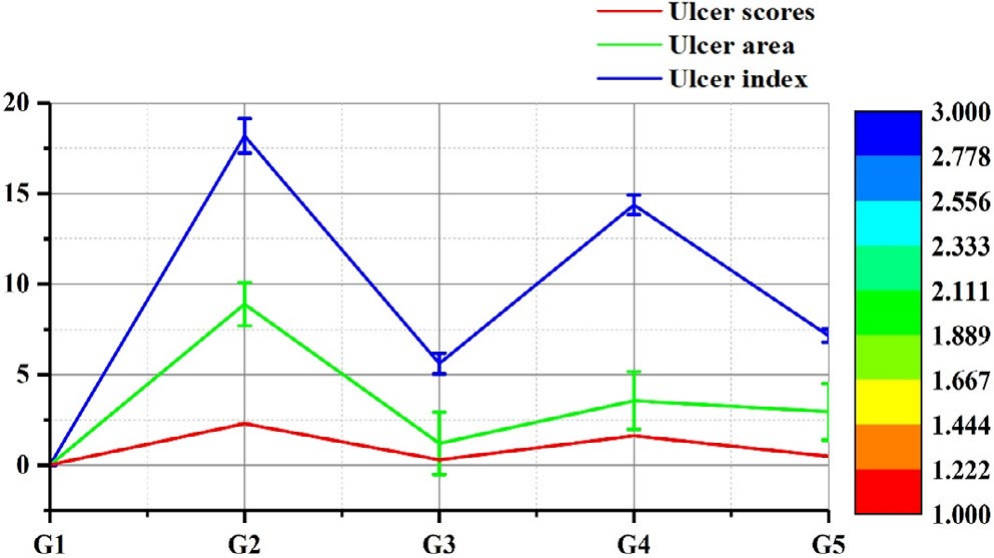
Mean ulcer scores, ulcer area (mm^2^), and ulcer index

Okra extract at the dosage of 250 and 500 mg/kg body weight significantly (*p* < .05) decreased the ulcer scoring than the aspirin‐treated group as presented in Figure 3. The highest ulcer score (2.29 ± 0.02) was observed in aspirin administrated rats G2 group, while the lowest ulcer score was observed in the control group G1 (0.03 ± 0.01). Okra extract at the dose of 500 mg/Kg of b.w showed better reduction (0.50 ± 0.06) in ulcer scores than 250 mg/Kg of b.w (1.62 ± 0.04). An increase in ulcer area (8.9 ± 1.2 mm^2^) was observed in the aspirin administrated group G2 than the control group G1 (0.01 ± 0.005 mm^2^). Omeprazole‐treated group G3 (1.2 ± 1.72 mm^2^) had the lowest ulcer area than others. Higher concentration of okra extract, 500 mg/Kg of b.w resulted in more reduction in ulcer area (2.95 ± 1.55 mm^2^) as compared to a lower dose of okra extract as shown in Figure 3. Anti‐ulcer drug omeprazole significantly (*p *< .05) decreased the ulcer index (5.61 ±** **0.58) followed by group G5 (7.15 ± 0.36) received okra extract dose 500 mg/Kg of b.w (Figure 3). An earlier study reported that the anti‐ulcer activity of methanolic and aqueous extracts of *Neolamarckia cadamba* bark and leaves in aspirin and pylorus ligation ulcer models. In both models, the common parameter determined was the ulcer index. Both extracts demonstrated the anti‐ulcerogenic activities by a reduction in ulcer index, which might be due to its antisecretory activity (Babu et al., 2014). In another study, Ortaç et al. (2018) reported that the okra extract 500 mg/kg of b.w resulted in a maximum reduction in ulcer index (5.26 ± 2.4) and it was even less than the reference drug famotidine (6.57 ± 2.7). Polyphenols and flavonoids like quercetin are important contributors to the gastroprotective action of okra (Gemede et al., 2016). The earlier investigation demonstrated that quercetin can protect DNA damage due to gastric mucosa cells infected by *H. pylori* (Klupinska et al., 2006). Ethanol‐induced gastric lesions are significantly inhibited by quercetin treatment (Abourehab et al., 2015). Gastric mucus content is increased by pretreatment with okra paste (Joshi et al., 2011). Quercetin can regulate immune cells’ invasion and can control the local immune response at the injury site and promoted wound healing by the modulation of TGF‐β1 and VEGF in the rat wounding model (Gopalakrishnan et al., 2016).

#### 3.3.5 Histopathological analysis

**FIGURE 4:**
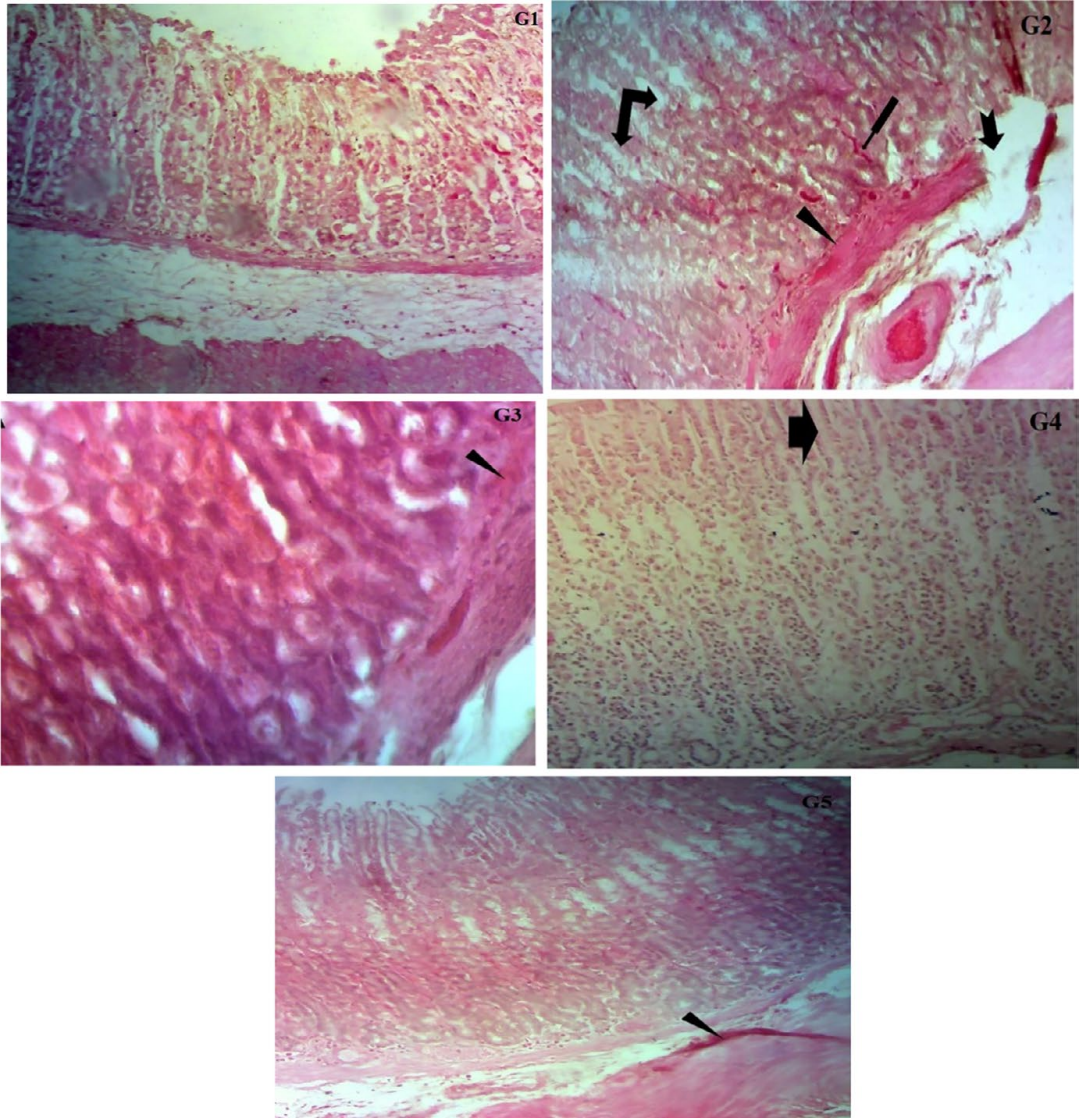
Histopathological analysis. Photomicrograph of rat stomach; Normal gastric mucosa (G1), congestion, gastric ulcer, muscular layer irregularity and damaged gastric pits (G2), congestion (G3), gastric pits increased in size (G4), congestion (G5). Congestion 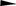 Gastric ulcer 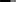 Muscular layer irregular 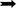 Gastric pits damaged 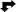 Gastric pits increased in size 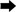

Photomicrographs of the cross‐sectional view of the rat stomach are shown in Figure 4. The normal architecture of gastric mucosa, submucosa, and muscular in G1 indicates regularity. The gastric epithelium is normal and intact. Gastric pits along with gastric glands also showed regularity. Small disruptions in the gastric epithelium layer were observed in gastric tissues of G2. Gastric pits increased in size and there was no regularity observed in them. Ulceration in the mucosal surface can also be seen along with congestion. Muscularis mucosa is not regular as well. The photomicrograph of rat stomach from group G3 depicts little congestion in the mucosal and muscular mucosal surface. However, gastric pits and gastric glands are normal and regular. Muscularis mucosa is also regular in a pattern. The microscopical view of tissues from G4 exhibited no sloughing and the epithelial layer remained undamaged. A small increase in gastric pits was observed. Gastric glands remained normal. Muscularis mucosa and gastric epithelium were also normal and regular, some infiltration of cells observed in the mucosa. The gastric mucosal layer remained undamaged with minor pathological modification or sloughing in gastric tissues of G5_._ Gastric pits are normal and regular, gastric glands show normal arrangement. The mucosal and submucosal surface is normal some congestion is also seen.

The results of the present investigation are in line with the earlier findings of Ortaç et al. (2018) who studied the anti‐ulcer genic effect of okra on ethanol‐induced acute gastric mucosal lesions in Wistar rats. Groups used in study were; okra 100, 250, 500 mg/kg, famotidine 20 mg/kg, quercetin 75 mg/kg and ethanol group. Okra 250, 500, and Fam 20 group showed decreased apoptosis, inflammatory changes, erosions, ulceration, and increased cell proliferation than the ethanol group. The nearly normal mucosal view was seen in okra 250 and 500 groups and effects were even comparable with the famotidine reference drug. Tissues from okra 100 exhibited little epithelial ulceration, hemorrhage, and edema (Ortaç et al., 2018). Quercetin combined with drug famotidine reduced the signs of hemorrhage and inflammation (Abourehab et al., 2015). In another investigation, the researchers explicated positive histopathological alterations in indomethacin intoxicated rats. Quercetin‐3‐O‐β‐D‐glucuronopyranoside attenuated indomethacin gastric mucosal damage due to increased gastric mucus secretion, decreased free radicals production due to activated neutrophils via ICAM‐1 and pro‐inflammatory cytokine down regulation (Gopalakrishnan et al., 2016).

## 4 CONCLUSION

The biochemical and histopathological explorations indicate that okra *A. esculentus* has gastroprotective potential against aspirin‐induced acute gastric lesions due to the presence of bioactive components. Okra helped in reducing congestion, irregularity in the muscular layer, increased gastric pits size, gastric ulcer area, ulcer scores, and ulcer area. Okra extract dose of 500 mg/Kg of body weight showed better results than 250 mg/Kg of body weight. Reduction in gastric ulcer by 500 mg/Kg of body weight was comparable to the drug omeprazole. However, more advanced research is required to highlight the fundamental mechanism of the gastroprotective potential of okra, to get more advantageous and fruitful outcomes in this respect.

## CONFLICT OF INTEREST

The authors declare no conflicts of interest.

## Data Availability

The dataset supporting the conclusions of this article is included within the article.
